# Knowledge, Attitudes, and Perceived Acceptability of Art Therapy Among Patients with Type 2 Diabetes Mellitus: A Cross-Sectional Survey in Nanjing, China

**DOI:** 10.3390/healthcare14152315

**Published:** 2026-07-31

**Authors:** Xuezhen Fu, Yangtian Wang, Li Yuan, Dan Xiang, Min Zhang, You Ge, Natalie Williamson, Weiming Tang, Shuaiju Liao

**Affiliations:** 1 School of Education, Johns Hopkins University, Baltimore, MD 21218, USA; xfu25@jh.edu; 2 Department of Endocrinology, Taikang Xianlin Drum Tower Hospital, Affiliated Hospital of the Medical School, Nanjing University, Nanjing 210046, China; wangyt66@tkhealthcare.com (Y.W.); yuanli28@tkhealthcare.com (L.Y.); xiangdan01@tkhealthcare.com (D.X.); zhangmin128@tkhealthcare.com (M.Z.); 3 Department of Science and Technology, The Second Hospital of Nanjing, Nanjing University of Chinese Medicine, Nanjing 210017, China; geyou_njey@njucm.edu.cn; 4 Institute for Global Health and Infectious Diseases, University of North Carolina at Chapel Hill, Chapel Hill, NC 27599, USA; natwill@unc.edu (N.W.); weiming_tang@med.unc.edu (W.T.)

**Keywords:** type 2 diabetes mellitus, art therapy, knowledge, attitude and practice, cross-sectional survey, complementary and alternative medicine

## Abstract

**Objective:** Art therapy has been proposed as a complementary approach to support psychosocial well-being in chronic disease management, but patients’ understanding and perceived acceptability of art therapy in type 2 diabetes mellitus (T2DM) remain unclear. This study aimed to describe item-level knowledge, attitudes, and art-related behaviors regarding art therapy among patients with T2DM in Nanjing, China, and to explore factors associated with willingness to try art therapy. **Methods:** A cross-sectional questionnaire survey was conducted from January to April 2025 among patients with T2DM receiving care at the Department of Endocrinology, Taikang Xianlin Drum Tower Hospital, Nanjing, China. Demographic characteristics and KAP related to art therapy for diabetes were collected. Univariable and multivariable logistic regression analyses were performed to explore factors associated with willingness to try art therapy. **Results:** A total of 982 patients with T2DM were included in the final analysis (mean age 50.61 ± 13.25 years; 662 (67.41%) male). Of these, 427 patients (43.48%) believed that art therapy is effective for diabetes, whereas 839 (85.44%) reported a willingness to try art therapy. In exploratory item-level analyses, patients who perceived art therapy as effective differed from those who did not across multiple knowledge, attitude, and behavior-related items. In multivariable logistic regression, lower willingness to try art therapy was associated with female sex (versus male, aOR = 3.914, 95%CI = 2.309–6.634), older age (aOR = 1.025 per year, 95%CI = 1.002–1.048), being a farmer or manual worker (versus teacher/cadre/medical staff, aOR = 2.129, 95%CI = 1.125–4.031), being unmarried/divorced (versus married, aOR = 9.961, 95% CI = 4.478–22.155), having out-of-province household registration (versus within province, aOR = 2.542, 95%CI = 1.333–4.850), being covered by employee/basic medical insurance (versus public expense or the new rural cooperative medical scheme, aOR = 1.961, 95%CI = 1.126–3.415), having chronic diseases (versus without chronic diseases, aOR = 2.495, 95% CI = 1.451–4.288), and frequent smoking (versus never smoking, aOR = 11.896, 95% CI = 5.555–25.477). **Conclusion:** Despite heterogeneous understanding of art therapy, most patients with T2DM reported willingness to try it, indicating generally favorable perceived acceptability. Future studies should examine how this stated willingness can be translated into informed participation in appropriately designed programs and evaluate their feasibility, uptake, and potential benefits.

## 1. Introduction

Type 2 diabetes mellitus (T2DM) is one of the leading chronic non-communicable diseases worldwide and accounts for more than 90% of all diabetes cases [1]. In China, the burden of T2DM remains substantial, and both treatment coverage and glycemic control remain suboptimal in many populations [2]. A nationally representative survey conducted in 2018 reported that only 32.9% of adults with diabetes were receiving treatment, and adequate glycemic control was achieved in only about half of treated patients [3]. Pharmacological therapy, lifestyle modification, regular glucose monitoring, and diabetes self-management education and support are the cornerstones of T2DM management according to national and international guidelines [4,5]. However, in real-world practice, many patients continue to experience treatment burden, suboptimal adherence, and diabetes-related complications despite standard care [6,7,8,9]. These complications include both macrovascular complications, such as cardiovascular disease, and microvascular complications, such as diabetic kidney disease, retinopathy, and neuropathy. In addition to physical complications, psychological distress is also common among patients with T2DM, including depression, anxiety, and diabetes-specific distress, which may further compromise self-management behaviors and glycemic outcomes [10,11]. These findings underscore the need for comprehensive management strategies that address not only biomedical indicators but also the psychological and behavioral dimensions of living with T2DM.

In addition to standard medical and behavioral management, complementary psychosocial approaches may have a supportive role in patient-centered diabetes care. Examples of supportive approaches include diabetes self-management education, psychological counseling, cognitive–behavioral strategies, and mindfulness-based interventions [12]. These approaches are not intended to replace pharmacological treatment or lifestyle modification, but rather to support emotional adjustment, self-efficacy, adherence, and long-term engagement with diabetes self-management [13]. Art therapy may be considered within this broader category of supportive psychosocial interventions [14], although it has been less frequently incorporated into routine diabetes care.

Art therapy is broadly defined as the use of artistic forms of expression as a complementary approach to disease management [15]. It integrates principles from psychology, visual and performing arts, and rehabilitation sciences, and can be delivered in individual or group settings under the guidance of trained professionals. Common modalities include visual art therapy (such as painting, drawing, and calligraphy), music therapy, and dance/movement therapy, as well as other creative practices that encourage symbolic expression and reflection [15,16]. By engaging patients in structured creative activities, art therapy may facilitate emotional expression, reduce perceived stress, enhance self-awareness and self-efficacy, strengthen therapeutic relationships, and foster social support, thereby helping patients cope with the emotional and behavioral burden of chronic illness [17,18,19].

Art therapy and related creative arts interventions have been explored in selected clinical populations, including patients with post-stroke depression, perinatal mood and anxiety disorders, postpartum depression, and post-traumatic stress disorder (PTSD) [20,21,22,23,24]. These studies provide broader contextual evidence regarding the potential psychosocial functions of creative arts interventions, but their findings cannot be directly extrapolated to patients with diabetes. The evidence specific to diabetes remains limited and heterogeneous. A previous meta-analysis focusing on patients with diabetes reported that art therapy was associated with improvements in depressive symptoms and blood glucose levels, but did not show significant effects on glycated hemoglobin or anxiety [14]. Individual studies of visual art therapy, guided imagery, and music-based interventions in patients with diabetes have reported selected improvements in emotional well-being, treatment-related behaviors, or quality of life, whereas findings for HbA1c and other glycemic outcomes have been mixed [25,26,27,28,29,30]. These findings suggest that art therapy may have potential value as a complementary psychosocial support approach, particularly for emotional distress and patient engagement. However, clinical evidence supporting the routine application of art therapy in diabetes care remains insufficient, possibly because of limited awareness among patients and healthcare providers, misconceptions about the need for artistic skill, insufficient availability of trained art therapists, and a lack of standardized diabetes-specific protocols [31,32]. Therefore, before considering broader implementation of art-based supportive programs, it is necessary to understand patients’ knowledge, attitudes, perceived acceptability, and existing art-related behaviors.

The knowledge-attitude-practice (KAP)-informed survey provides a practical approach to identifying knowledge gaps, attitudes, behavioral patterns, and potential barriers related to emerging health-related interventions [33]. Because art therapy remains unfamiliar to many patients with T2DM in China, such a survey may help clarify patients’ understanding of art therapy, their attitudes toward its potential role in diabetes care, their willingness to try art-based supportive activities, and the factors associated with perceived acceptability. Accordingly, this exploratory study aimed to address two research questions: (1) how do patients with T2DM understand and perceive art therapy and related art-based supportive activities; and (2) which patient characteristics are associated with unwillingness to try art therapy? In the present study, we used item-level KAP information to describe patients’ understanding of art therapy, their attitudes toward its potential role in diabetes care, their perceived acceptability and willingness to try it, and their art-related behaviors or hobbies.

## 2. Methods

### 2.1. Study Participants

From January to April 2025, we conducted a single-center, cross-sectional survey among patients with T2DM who were receiving care at the Department of Endocrinology, Taikang Xianlin Drum Tower Hospital, Nanjing, China. Participants were recruited using convenience sampling. During the study period, eligible patients who attended the department and were willing to participate were invited to complete the questionnaire. Eligible participants met the following criteria: (1) age ≥ 18 years; (2) confirmed diagnosis of T2DM; and (3) capacity to provide informed consent and willingness to participate. Patients with type 1 diabetes or other specific types of diabetes were excluded to maintain clinical homogeneity. This study was approved by the Ethics Committee of Taikang Xianlin Drum Tower Hospital (Approval No. 2025-014-01). All participants provided written informed consent.

### 2.2. Questionnaire Survey and Data Collection

A self-designed, item-level knowledge-attitude-practice (KAP) questionnaire was developed for this study based on the Chinese Guidelines for the Prevention and Treatment of Type 2 Diabetes Mellitus (2024 Edition), relevant concepts of art therapy, and expert input from diabetes management and psychology. The questionnaire was designed for descriptive item-level analysis and was not intended to generate a standardized total KAP or domain score. The items assessed several related but distinct concepts and included binary, categorical, and conditional multiple-response formats. They were intended to be interpreted individually rather than as interchangeable indicators of a single latent construct. Therefore, internal consistency coefficients, factor-structure analyses, and scale-score analyses were not prespecified. In addition, convergent validity could not be assessed because no established validated instrument measuring the same construct was administered in the present survey. In this questionnaire-based survey, art therapy was operationalized as a broad patient-facing concept. We distinguished professional art therapy, which refers to a structured therapeutic approach using artistic expression and psychological principles, from art-based supportive activities and personal art-related hobbies. Accordingly, the questionnaire assessed three related but distinct aspects: patients’ knowledge of professional art therapy, their attitudes toward art therapy and art-based supportive activities that may be considered in diabetes care, and their existing art-related behaviors or hobbies. This study did not evaluate the clinical effectiveness of professional art therapy.

The questionnaire comprised four domains: (1) general sociodemographic and clinical characteristics; (2) knowledge of art therapy and its perceived role in disease management; (3) attitudes toward art therapy, perceived acceptability, and willingness to try art therapy or related art-based supportive activities; and (4) art-related behaviors, hobbies, and engagement in art-related activities after the diagnosis of diabetes (Supplementary Table S1). Data were collected on site through face-to-face, investigator-assisted interviews. With guidance from trained investigators, participants completed an electronic questionnaire via the online “Questionnaire Star” platform, which was used only as an electronic data-entry tool. For participants who were unable to use a smartphone, an equivalent paper-based version was administered.

### 2.3. Quality Control

The draft questionnaire was reviewed by experts in diabetes management and psychology to assess content relevance, clarity, and appropriateness for patients with T2DM. A pilot survey was then conducted among a small group of patients with T2DM to evaluate the comprehensibility, feasibility, and completion time of the questionnaire. Based on expert and patient feedback, minor revisions were made, including clarification of conditional questions and refinement of several response options. Prior to the formal survey, all investigators received standardized training on the study objectives, questionnaire content, and survey procedures. During data collection, a designated quality controller systematically reviewed all completed questionnaires. Forms with missing or inconsistent information were flagged, and participants were contacted when necessary to clarify or supplement the data.

### 2.4. Sample Size

The sample size was primarily determined for the descriptive objective of this cross-sectional survey using the formula for estimating a single population proportion:

$$n\;=\;\frac{Z_{\alpha /2}^{2}p(1\;-\;p)}{d^{2}}$$

where $Z_{\alpha /2}\;=\;1.96$ for a two-sided 95% confidence level, p was the expected proportion, and d was the allowable margin of error. In the pilot survey, 83.3% of participants reported willingness to try art therapy. Using p = 0.833 and d = 0.05, the minimum required sample size was calculated to be 214. After allowing for a 20% non-response or invalid-response rate, the target sample size was increased to 268. The final analytic sample comprised 982 participants, exceeding the prespecified requirement.

For the exploratory multivariable logistic regression analysis, model adequacy was additionally considered according to the number of outcome events relative to the number of estimated parameters. Unwillingness to try art therapy was coded as the outcome event, and 143 participants reported unwillingness. The final multivariable model corresponds to approximately 10 outcome events per parameter.

### 2.5. Statistical Analysis

Categorical variables were summarized as frequencies and percentages. Continuous variables were expressed as mean ± standard deviation (SD). Participants were stratified according to their perceived effectiveness of art therapy for diabetes. This grouping variable was derived from the questionnaire item assessing whether participants believed that art therapy was effective for diabetes (Supplementary Table S1). Participants who perceived art therapy as effective were classified into the “art therapy effective” group, whereas those who did not perceive it as effective or were uncertain were classified into the “art therapy not effective” group. Between-group comparisons were performed using Pearson’s chi-square test or Fisher’s exact test for categorical variables, as appropriate. Continuous variables were compared using two-sample t-test. Given the large number of exploratory between-group comparisons, the Benjamini–Hochberg procedure was applied to control the false discovery rate (FDR). Adjustment was performed separately within each analytical domain: knowledge items, attitude items, and behavior-related items. A q value < 0.05 was considered statistically significant after FDR adjustment. Univariable and multivariable logistic regression analyses were conducted to identify factors associated with unwillingness to try art therapy. The variable of willingness to try art therapy was derived from a single binary questionnaire item asking whether the participant was willing to try art therapy (Supplementary Table S1). Responses were coded as 1 for “no” and 0 for “yes” in the logistic regression analyses. As the logistic regression analysis was exploratory, no interaction terms were prespecified or tested in the regression model. A two-sided *p* value < 0.05 was considered statistically significant. Data were managed and analyzed using R software (version 4.2.1).

## 3. Results

### 3.1. Characteristics of the Participants

Of the 1061 patients with diabetes were surveyed, 74 patients with type 1 diabetes mellitus and 5 patients with gestational diabetes mellitus were excluded. Consequently, 982 patients with type 2 diabetes mellitus were included in the final analysis. The mean age was 50.61 ± 13.25 years, and 662 (67.41%) were male. Overall, 702 participants (71.49%) had attained a college-level education or higher, 896 (91.24%) were married, and 894 (91.04%) held local provincial household registration. When patients who believed that art therapy is effective for diabetes were compared with those who did not, statistically significant differences were observed in sex, occupation, height, weight, education level, household registration, monthly income, presence of infectious diseases, smoking status, and alcohol consumption (all *p* < 0.05). Detailed characteristics are presented in Table 1.

### 3.2. Knowledge Related to Art Therapy

Among the 982 participants, 786 (80.29%) agreed that “art therapy can play an auxiliary role in the treatment of diseases,” and 427 (43.48%) believed that “art therapy is effective in the treatment of diabetes.” A total of 200 participants (20.37%) thought that “any healthcare worker can provide art therapy,” whereas 133 (13.54%) incorrectly believed that “art therapy can replace medication.” When patients were stratified according to whether they believed art therapy is effective for diabetes, statistically significant differences were observed between groups for nearly all knowledge items, with the exception of the statements “The main tools of art therapy include eight forms of art such as painting and calligraphy” and “Art therapy can replace medication” (all other q < 0.05) (Table 2).

### 3.3. Attitude Toward Art Therapy

Among the 982 participants, 839 (85.44%) reported that they were willing to try art therapy. The main reasons for willingness included perceiving it as a new experience (91.42%), believing it to be effective (92.37%), encouragement from healthcare professionals (62.22%), and encouragement from family members (7.15%). Reasons for reluctance to try art therapy included psychological resistance (34.27%), perceiving it as ineffective (56.85%), disliking art (50.35%), and having no artistic hobbies (49.65%). A total of 874 participants (89.00%) believed that “art therapy can assist in the treatment of diabetes,” and they most commonly cited psychological comfort (78.60%), relief of tension (75.63%), distraction of attention (66.13%), and enhanced interaction with healthcare professionals (38.90%) as its main benefits. Among those who believed that art therapy cannot assist in treating diabetes, the most frequently cited reasons were a lack of knowledge about art therapy (100.00%), a perception that diabetes is an organic disease (60.55%), and difficulty in evaluating the effectiveness of art therapy (54.63%). When participants who believed art therapy is effective for diabetes were compared with those who did not, between-group differences were observed in selected reasons for willingness, preferred forms of art therapy among participants willing to try it, having no artistic hobbies as a reason for unwillingness, belief in the auxiliary role of art therapy in diabetes treatment, several perceived functions of art therapy, selected preferences for painting styles, and preferred content for waiting-room screens (all q < 0.05). In contrast, the between-group difference in overall willingness to try art therapy did not remain statistically significant after FDR adjustment (raw *p* = 0.041; q = 0.064). Detailed results are presented in Table 3.

### 3.4. Behavioral Characteristics

Among the 982 participants, 613 (62.42%) reported that their treatment consisted mainly of insulin injections or oral hypoglycemic agents. Fasting blood glucose levels were between 7.0 and 8.0 mmol/L in 550 patients (56.01%). A total of 763 participants (77.70%) were engaged in light physical work. Following the diagnosis of diabetes, 97.76% of patients reported reducing their staple food intake, and 55.84% indicated that their physicians had advised them to cultivate an artistic hobby. When participants were stratified according to whether they believed art therapy was effective in diabetes treatment, statistically significant differences were observed in the number of oral hypoglycemic agents used, fasting blood glucose level, type of occupation, main form of exercise, dietary modifications, and whether healthcare workers had recommended developing an artistic hobby after diagnosis (all q < 0.05). Detailed results are presented in Table 4.

### 3.5. Factors Associated with Unwillingness to Try Art Therapy

Willingness to try art therapy was treated as the dependent variable, and patients’ basic characteristics were entered as independent variables. Univariable and multivariable logistic regression analyses were performed to identify factors associated with unwillingness to use art therapy. Multicollinearity diagnostics showed no evidence of problematic collinearity among the predictors, with all adjusted GVIF values below 1.40. The model was statistically significant overall (likelihood-ratio χ^2^ = 227.50, *p* < 0.001) and showed acceptable calibration (Hosmer–Lemeshow χ^2^ = 15.33, *p* = 0.053). Its Brier score was 0.092, and the area under the receiver operating characteristic curve was 0.858 (95% CI: 0.829–0.888), indicating good discrimination. Detailed model performance measures are presented in Supplementary Table S2. In univariable analyses, unwillingness was significantly associated with sex, age, occupation, education level, marital status, household registration, monthly income, type of medical insurance, presence of chronic diseases, smoking, and alcohol consumption. Multivariable logistic regression showed that female sex, older age, working as a farmer or manual worker, being unmarried/divorced, having non-local (out-of-province) household registration, being covered by employee/basic medical insurance, having chronic comorbidities, and frequent smoking were independently associated with unwillingness to try art therapy (all *p* < 0.05). Detailed results are presented in Table 5.

## 4. Discussion

Art therapy, which emerged as an interdisciplinary field in Western countries after World War II, is grounded in psychology, art theory, sociology, and other related disciplines. It has been used globally as a complementary and alternative treatment for a broad range of physical and mental health conditions [15]. In China, the implementation of art therapy began in the 1990s and has gradually expanded within both educational and medical settings [34]. However, evidence regarding its effectiveness in T2DM remains limited and heterogeneous. Earlier studies included individual visual art therapy for youth with poorly controlled T1DM, auditory guided imagery accompanied by music, therapist-delivered music therapy, and structured music-listening programs [25,26,27]. These studies reported selected improvements in behavioral difficulties, anxiety, depression, and stress. Similarly, more recent studies of art therapy in adolescents with T1DM and older adults with T2DM have provided additional evidence of potential benefits for emotional well-being, cognition, and quality of life [28,29,30]. However, improvements in HbA1c and other glycemic outcomes in these studies have generally been inconsistent. Taken together, the available evidence suggests that art therapy may have value as an adjunctive psychosocial approach by facilitating emotional expression, reducing psychological distress, and supporting patients’ engagement in diabetes self-management; however, the current evidence does not establish its clinical effectiveness in patients with T2DM. These findings provide a rationale for examining whether patients with T2DM understand, accept, and are willing to engage with such supportive approaches.

To the best of our knowledge, this is the first questionnaire-based survey to systematically describe item-level knowledge, attitudes, perceived acceptability, and art-related behaviors concerning art therapy among patients with T2DM. The findings revealed a distinctive pattern of uneven understanding accompanied by broadly favorable attitudes. Fewer than half of the participants considered art therapy to be effective specifically for diabetes, suggesting that confidence in its therapeutic value was not universal. Nevertheless, nearly 90% believed that it could play an auxiliary role in diabetes treatment, and 85.44% expressed willingness to try it. Taken together, these results suggest that many patients may remain cautious about making a strong judgment regarding effectiveness while still being receptive to art therapy as a complementary form of psychosocial support. The reasons reported by willing participants further illustrate this openness. Novelty and perceived effectiveness were the most frequently selected reasons for willingness, indicating that patients may be attracted both by the opportunity to experience a different form of supportive care and by an expectation that it may provide some benefit. Painting was among the most widely accepted forms, although preferences also extended to calligraphy, music, drama, and other creative activities. These preferences provide useful information for the design of future programs because the acceptability of art-based interventions may depend not only on their therapeutic rationale but also on whether their formats are accessible, culturally familiar, and consistent with patients’ interests.

An important finding was that approximately 85% of participants reported having no existing art-related hobbies, despite the high proportion who were willing to try art therapy. This contrast suggests that openness to art therapy does not necessarily depend on previous artistic experience. Formal participation in art therapy generally does not require prior artistic training or technical ability, and patients without established hobbies may still be receptive when activities are appropriately introduced and supported. At the same time, limited previous engagement may reflect a lack of exposure, opportunity, time, or accessible settings in which patients can participate in creative activities.

The multivariable analysis further demonstrated that willingness was not evenly distributed across patient groups. Female sex, older age, being a farmer or manual worker, belonging to the combined unmarried/divorced category, having out-of-province household registration, being covered by employee/basic medical insurance, having chronic diseases, and frequent smoking were associated with higher odds of unwillingness to try art therapy. Notably, the particularly strong association observed between frequent smoking and unwillingness to try art therapy should be interpreted with caution. Smoking frequency was self-reported and did not capture smoking intensity or nicotine dependence. Therefore, this finding should not be interpreted as evidence that frequent smoking directly reduces willingness to engage in art therapy. Rather, frequent smoking may have served as a proxy for unmeasured behavioral, psychosocial, socioeconomic, or healthcare-engagement characteristics. In addition, the relatively wide confidence interval indicates considerable uncertainty regarding the magnitude of the association. Further studies are therefore needed to assess the robustness and replicability of this finding. These findings suggest that perceived acceptability may be influenced by patients’ broader social circumstances, available resources, health burden, and prior exposure to psychosocial or complementary services. Patient education and service delivery may need to be adapted according to age, occupation, health status, social support, and practical accessibility. Qualitative interviews could be used to explore why some patient groups are less willing, whether their concerns relate to unfamiliarity, perceived relevance, time burden, artistic confidence, or other barriers, and what forms of delivery they would consider acceptable.

This study has several limitations. First, its cross-sectional design does not permit determination of temporal or causal relationships. Several potentially relevant clinical and psychosocial variables, including HbA1c, diabetic complications, diabetes-related distress, and history of psychological disorders, were not systematically collected. These factors may be associated with the perceived acceptability of psychosocial interventions and may have partly contributed to the observed associations. Therefore, the regression findings should be interpreted as exploratory, and residual confounding cannot be excluded. Second, participants were recruited using convenience sampling from a single hospital, which may have introduced selection bias. Moreover, 71.49% of participants had attained a college-level education or above. The study sample should therefore be regarded as a relatively highly educated, hospital-based clinical population rather than as representative of the broader population of people with T2DM in China. Consequently, the observed level of willingness and the associated factors may differ across regions, healthcare settings, and populations with different educational backgrounds. Future multicenter studies using more representative sampling strategies are needed to assess the generalizability of these findings. Third, the questionnaire was self-developed for descriptive item-level analysis rather than as a standardized psychometric scale. Because the items covered heterogeneous concepts and response formats, they were analyzed individually rather than combined into scale scores. Although expert review and pilot testing improved clarity and feasibility, formal psychometric properties, including internal consistency, factor structure, and convergent validity, were not evaluated. This may limit the reproducibility of our findings and their comparability across studies. Future research should develop and formally validate a standardized instrument for assessing knowledge and perceived acceptability of art therapy among T2DM patients. Moreover, art therapy was operationalized broadly and included items relating to professional art therapy, art-based supportive activities, and personal artistic hobbies. Participants may have interpreted the term “art therapy” inconsistently. The willingness outcome was also assessed using a single binary item and therefore represented stated acceptability rather than a validated behavioral measure. Finally, the data were self-reported and collected through investigator-assisted questionnaires, which may have introduced recall bias, social desirability bias, and response bias, particularly for questions concerning willingness and health-related behaviors.

## 5. Conclusions

This study found that patients with T2DM showed a heterogeneous understanding of art therapy but had generally favorable attitudes toward its potential supportive role, with most participants reporting a willingness to try it. Differences in willingness across sociodemographic and health-related groups indicate that program information and delivery may need to be tailored to patients’ practical circumstances and preferences. Future studies should determine whether the stated willingness translates into actual uptake, sustained participation, and meaningful psychosocial or clinical outcomes.

## Figures and Tables

**Table 1:** Baseline characteristics of patients with T2DM.

Variable		Total Patients	Art Therapy Effective	Art Therapy Not Effective	*p* Value
Gender					<0.001
	Male	662 (67.41)	319 (74.71)	343 (61.80)	
	Female	320 (32.59)	108 (25.29)	212 (38.20)	
Age (years)		50.61 ± 13.25	49.78 ± 13.18	51.25 ± 13.28	0.087
Occupation					<0.001
	Teacher/cadre/medical staff	363 (36.97)	188 (44.03)	175 (31.53)	
	Farmer/manual worker	214 (21.79)	73 (17.10)	141 (25.41)	
	Student/catering & food industry/commercial service/housework & unemployed/self-employed/other	405 (41.24)	166 (38.88)	239 (43.06)	
Height (cm)		168.99 ± 6.61	169.64 ± 6.57	168.49 ± 6.61	0.006
Weight (kg)		71.30 ± 9.28	71.45 ± 7.09	71.18 ± 10.67	0.004
Education level					<0.001
	College or above	702 (71.49)	358 (83.84)	344 (61.98)	
	High school or below	280 (28.51)	69 (16.16)	211 (38.02)	
Marital status					0.092
	Married	896 (91.24)	397 (92.97)	499 (89.91)	
	Unmarried/divorced	86 (8.76)	30 (7.03)	56 (10.09)	
Household registration					<0.001
	Within province	894 (91.04)	405 (94.85)	489 (88.11)	
	Outside province	88 (8.96)	22 (5.15)	66 (11.89)	
Monthly income (RMB)					<0.001
	<5000	195 (19.86)	77 (18.03)	118 (21.26)	
	5000–9999	596 (60.69)	230 (53.86)	366 (65.95)	
	≥10,000	191 (19.45)	120 (28.10)	71 (12.79)	
Type of medical insurance					0.509
	Employee/basic medical insurance	746 (75.97)	320 (74.94)	426 (76.76)	
	Public expense/rural cooperative	236 (24.03)	107 (25.06)	129 (23.24)	
Diabetes duration (years)					0.627
	≤5	441 (44.91)	188 (44.03)	253 (45.59)	
	>5	541 (55.09)	239 (55.97)	302 (54.51)	
Current chronic diseases					
	Hypertension (Yes)	512 (52.14)	226 (52.93)	286 (51.53)	0.664
	Hyperlipidemia (Yes)	291 (29.63)	122 (28.57)	169 (30.45)	0.523
	Coronary heart disease (Yes)	77 (7.84)	35 (8.20)	42 (7.57)	0.716
	None of the above	374 (38.09)	148 (34.66)	226 (40.72)	0.053
Infectious diseases					0.032
	No	962 (97.96)	423 (99.06)	539 (97.12)	
	Yes	20 (2.04)	4 (0.94)	16 (2.88)	
Smoking frequency					<0.001
	Never	399 (40.63)	149 (34.89)	250 (45.05)	
	Occasionally	485 (49.39)	219 (51.29)	266 (47.93)	
	Frequently	98 (9.98)	59 (13.82)	39 (7.03)	
Alcohol drinking frequency					<0.001
	Never	251 (25.56)	103 (24.12)	148 (26.67)	
	Occasionally	529 (53.87)	211 (49.41)	318 (57.30)	
	Frequently	202 (20.57)	113 (26.46)	89 (16.04)	

**Table 2:** Knowledge of art therapy among patients with T2DM.

Variable	Total Patients	Art Therapy Effective	Art Therapy Not Effective	*p* Value	Q Value
Art therapy is a comprehensive treatment method that combines expressive arts and psychology				<0.001	<0.001
Yes	754 (76.78)	369 (86.42)	385 (69.37)		
No	228 (23.22)	58 (13.58)	170 (30.63)		
Art therapy refers to the impact of artistic forms of expression projected by patients				<0.001	<0.001
Yes	377 (38.39)	270 (63.23)	107 (19.28)		
No	605 (61.61)	157 (36.77)	448 (80.72)		
Art therapy can play an auxiliary role in the treatment of diseases				<0.001	<0.001
Yes	786 (80.29)	378 (89.15)	408 (73.51)		
No	193 (19.71)	46 (10.85)	147 (26.49)		
The main tools of art therapy include eight forms of art such as painting and calligraphy				0.114	0.114
Yes	218 (22.20)	105 (24.59)	113 (20.36)		
No	764 (77.80)	322 (75.41)	442 (79.64)		
Art therapy can replace medication				0.085	0.102
Yes	133 (13.54)	67 (15.69)	66 (11.89)		
No	849 (86.46)	360 (84.31)	489 (88.11)		
Any healthcare worker can provide art therapy				0.004	0.006
Yes	200 (20.37)	105 (24.59)	95 (17.12)		
No	782 (79.63)	322 (75.41)	460 (82.88)		

**Table 3:** Attitude toward art therapy among patients with T2DM.

Variable	Total Patients	Art Therapy Effective	Art Therapy Not Effective	*p* Value	Q Value
Willingness to try art therapy				0.041	0.064
No	143 (14.56)	51 (11.94)	92 (16.58)		
Yes	839 (85.44)	376 (88.06)	463 (83.42)		
Reasons for willingness to try art therapy (N = 839)					
Perceiving it as a new experience				0.011	0.020
No	72 (8.58)	22 (5.85)	50 (10.80)		
Yes	767 (91.42)	354 (94.15)	413 (89.20)		
Believing it to be effective				0.045	0.054
No	64 (7.63)	21 (5.59)	43 (9.29)		
Yes	775 (92.37)	355 (94.41)	420 (90.71)		
Encouragement from healthcare professionals				0.150	0.165
No	317 (37.78)	132 (35.11)	185 (39.96)		
Yes	522 (62.22)	244 (64.89)	278 (60.04)		
Encouragement from family members				0.765	0.765
No	779 (92.85)	348 (92.55)	431 (93.09)		
Yes	60 (7.15)	28 (7.45)	32 (6.91)		
Others				<0.001	0.001
No	823 (98.09)	376 (100.00)	447 (96.54)		
Yes	16 (1.91)	0 (0.00)	16 (3.46)		
Preferred forms of art therapy if tried (N = 839)					
Painting				0.013	0.021
No	232 (27.65)	88 (23.40)	144 (31.10)		
Yes	607 (72.35)	288 (76.60)	319 (68.90)		
Calligraphy				<0.001	<0.001
No	430 (51.25)	156 (41.49)	274 (59.18)		
Yes	409 (48.75)	220 (58.51)	189 (40.82)		
Drama/theatre				0.016	0.022
No	458 (54.59)	188 (50.00)	270 (58.32)		
Yes	381 (45.41)	188 (50.00)	193 (41.68)		
Art salon				0.005	0.010
No	467 (55.66)	189 (50.27)	278 (60.04)		
Yes	372 (44.34)	187 (49.73)	185 (39.96)		
Music				<0.001	<0.001
No	409 (48.75)	220 (58.51)	189 (40.82)		
Yes	430 (51.25)	156 (41.49)	274 (59.18)		
Others				<0.001	0.001
No	809 (96.42)	372 (98.94)	437 (94.38)		
Yes	30 (3.58)	4 (1.06)	26 (5.62)		
Reasons for unwillingness to try art therapy (N = 143)					
Psychological resistance				0.096	0.160
No	94 (65.73)	29 (56.86)	65 (70.65)		
Yes	49 (34.27)	22 (43.14)	27 (29.35)		
Perceiving it as ineffective				0.023	0.057
No	63 (44.06)	16 (31.37)	47 (51.09)		
Yes	80 (55.94)	35 (68.63)	45 (48.91)		
Disliking art				0.813	0.813
No	71 (49.65)	26 (50.98)	45 (48.91)		
Yes	72 (50.35)	25 (49.02)	47 (51.09)		
Having no artistic hobbies				0.001	0.006
No	72 (50.35)	35 (68.63)	37 (40.22)		
Yes	71 (49.65)	16 (31.37)	55 (59.78)		
Others				0.304	0.380
No	117 (81.82)	44 (86.27)	73 (79.35)		
Yes	26 (18.18)	7 (13.73)	19 (20.65)		
Belief that art therapy can assist in treating diabetes				<0.001	<0.001
No	108 (11.00)	20 (4.68)	88 (15.86)		
Yes	874 (89.00)	407 (95.32)	467 (84.14)		
Perceived main functions of art therapy in assisting diabetes treatment (N = 874)					
Psychological comfort				0.003	0.007
No	187 (21.40)	69 (16.95)	118 (25.27)		
Yes	687 (78.60)	338 (83.05)	349 (74.73)		
Relief of tension				0.004	0.007
No	213 (24.37)	81 (19.90)	132 (28.27)		
Yes	661 (75.63)	326 (80.10)	335 (71.73)		
Distraction of attention				0.033	0.042
No	296 (33.87)	123 (30.22)	173 (37.04)		
Yes	578 (66.13)	284 (69.78)	294 (62.96)		
Promoting interaction with healthcare professionals				<0.001	0.005
No	534 (61.10)	225 (55.28)	309 (66.17)		
Yes	340 (38.90)	182 (44.72)	158 (33.83)		
Others				0.355	0.355
No	862 (98.63)	403 (99.02)	459 (98.29)		
Yes	12 (1.37)	4 (0.98)	8 (1.71)		
Reasons for believing art therapy cannot assist in treating diabetes (N = 108)					
Not familiar with art therapy				1.000	1.000
No	0 (0.00)	0 (0.00)	0 (0.00)		
Yes	108 (100.00)	20 (100.00)	88 (100.00)		
Belief that diabetes is an organic disease				<0.001	<0.001
No	43 (39.81)	0 (0.00)	43 (48.86)		
Yes	65 (60.19)	20 (100.00)	45 (51.14)		
Difficulty in evaluating effectiveness				0.126	0.189
No	49 (45.37)	6 (30.00)	43 (48.86)		
Yes	59 (54.63)	14 (70.00)	45 (51.14)		
Preferred painting styles as a medium for art therapy					
Chinese painting	717 (73.01)	342 (80.09)	375 (67.57)	<0.001	<0.001
Oil painting	522 (53.16)	253 (59.25)	269 (48.47)	<0.001	0.002
Watercolor/pastel	351 (35.74)	133 (31.15)	218 (39.28)	0.008	0.016
Sketch	464 (47.25)	187 (43.79)	277 (49.91)	0.057	0.080
Others	16 (1.63)	0 (0.00)	16 (2.88)	<0.001	0.001
Preference during painting-based art therapy				0.209	0.222
Viewing others’ paintings	125 (12.73)	46 (10.77)	79 (14.23)		
Painting by oneself	215 (21.89)	91 (21.31)	124 (22.34)		
Both viewing and painting	642 (65.38)	290 (67.92)	352 (63.42)		
Preferred paintings in hospital corridors					
Landscape paintings	768 (78.21)	345 (80.80)	423 (76.22)	0.085	0.103
Flower-and-bird paintings	693 (70.57)	306 (71.66)	387 (69.73)	0.510	0.510
Portraits of famous people	464 (47.25)	185 (43.33)	279 (50.27)	0.031	0.052
Preferred content on waiting-room screens (beyond calling numbers)					
Movies	550 (56.01)	206 (48.24)	344 (61.98)	<0.001	<0.001
Short videos	700 (71.28)	328 (76.81)	372 (67.03)	<0.001	0.002
Cartoons	747 (76.07)	360 (84.31)	387 (69.73)	<0.001	<0.001
Medical advertisements	192 (19.55)	92 (21.55)	100 (18.02)	0.167	0.189
Others	20 (2.04)	0 (0.00)	20 (3.60)	<0.001	<0.001
Acceptable ways for non-art-therapist physicians to be involved in art therapy				0.061	0.080
Encouraging patients to undertake art therapy	666 (67.82)	276 (64.64)	390 (70.27)		
Directly delivering art therapy to patients	316 (32.18)	151 (35.36)	165 (29.73)		

**Table 4:** Diabetes-related behaviors and art-related hobbies among patients with T2DM.

Variable	Total Patients	Art Therapy Effective	Art Therapy Not Effective	*p* Value	Q Value
Type of medication currently used for diabetes				0.952	0.952
Insulin injections or oral hypoglycemic agents	613 (62.42)	267 (62.53)	346 (62.34)		
Insulin injections plus oral hypoglycemic agents	369 (37.58)	160 (37.47)	209 (37.66)		
Number of oral hypoglycemic agents currently taken				<0.001	0.001
1	89 (9.41)	32 (7.73)	57 (10.71)		
2	555 (58.67)	272 (65.70)	283 (53.20)		
≥3	302 (31.92)	110 (26.57)	192 (36.09)		
Fasting blood glucose level				0.012	0.020
<7.0	228 (23.22)	117 (27.40)	111 (20.00)		
7.0–8.0	550 (56.01)	219 (51.29)	331 (59.64)		
>8.0	204 (20.77)	91 (21.31)	113 (20.36)		
Type of work					
Light physical work	763 (77.70)	331 (77.52)	432 (77.84)	0.905	0.941
Heavy physical work	6 (0.61)	6 (1.41)	0 (0.00)	0.007	0.012
Mental work	218 (22.20)	101 (23.65)	117 (21.08)	0.336	0.380
Other	18 (1.83)	0 (0.00)	18 (3.24)	<0.001	<0.001
Main type of exercise before diabetes diagnosis					
Walking	915 (93.18)	406 (95.08)	509 (91.71)	0.038	0.058
Jogging	810 (82.48)	387 (90.63)	423 (76.22)	<0.001	<0.001
Swimming	417 (42.46)	212 (49.65)	205 (36.94)	<0.001	<0.001
Square dancing	44 (4.48)	23 (5.39)	21 (3.78)	0.229	0.283
Others	11 (1.12)	4 (0.94)	7 (1.26)	0.765	0.828
No exercise	34 (3.46)	0 (0.00)	34 (6.13)	<0.001	<0.001
Main dietary changes after diabetes diagnosis					
Reduced staple foods	960 (97.76)	427 (100.00)	533 (96.04)	<0.001	<0.001
Reduced meat intake	847 (86.25)	386 (90.40)	461 (83.06)	<0.001	0.002
Increased vegetable intake	782 (79.63)	318 (74.47)	464 (83.60)	<0.001	0.001
Increased water intake	442 (45.01)	201 (47.07)	241 (43.42)	0.255	0.301
Increased fish consumption	170 (17.31)	64 (14.99)	106 (19.10)	0.091	0.121
Others	26 (2.65)	4 (0.94)	22 (3.96)	0.003	0.007
Recommendation from healthcare workers to develop an artistic hobby after diagnosis				0.008	0.014
No	367 (44.16)	193 (48.98)	174 (39.82)		
Yes	464 (55.84)	201 (51.02)	263 (60.18)		
Art-related hobbies					
Painting	53 (5.40)	9 (2.11)	44 (7.93)	<0.001	<0.001
Calligraphy	57 (5.80)	8 (1.87)	49 (8.83)	<0.001	<0.001
Drama	17 (1.73)	1 (0.23)	16 (2.88)	0.002	0.003
Singing/musical instruments	91 (9.27)	32 (7.49)	59 (10.63)	0.093	0.121
Others	23 (2.34)	6 (1.41)	17 (3.06)	0.089	0.121
No art-related hobby	831 (84.62)	394 (92.27)	437 (78.74)	<0.001	<0.001
Type of painting medium skilled at (N = 53)					
Chinese painting				0.536	0.715
No	4 (7.55)	1 (11.11)	3 (6.82)		
Yes	49 (92.45)	8 (88.89)	41 (93.18)		
Oil painting				0.242	0.485
No	36 (67.92)	8 (88.89)	28 (63.64)		
Yes	17 (32.08)	1 (11.11)	16 (36.36)		
Watercolor/pastel				0.715	0.817
No	30 (56.60)	6 (66.67)	24 (54.55)		
Yes	23 (43.40)	3 (33.33)	20 (45.45)		
Sketch				0.005	0.019
No	18 (33.96)	7 (77.78)	11 (25.00)		
Yes	35 (66.04)	2 (22.22)	33 (75.00)		
Others				1.000	1.000
No	53 (100.00)	9 (100.00)	44 (100.00)		
Yes	0 (0.00)	0 (0.00)	0 (0.00)		
Main type of painting usually created					
Landscape/scenery				0.313	0.502
No	2 (3.77)	1 (11.11)	1 (2.27)		
Yes	51 (96.23)	8 (88.89)	43 (97.73)		
Flower-and-bird/still life				0.076	0.204
No	26 (49.06)	7 (77.78)	19 (43.18)		
Yes	27 (50.94)	2 (22.22)	25 (56.82)		
Portraits				0.002	0.014
No	26 (49.06)	0 (0.00)	26 (59.09)		
Yes	27 (50.94)	9 (100.00)	18 (40.91)		

**Table 5:** Univariable and multivariable logistic regression analyses of factors associated with unwillingness to try art therapy among patients with T2DM.

Variable		Univariable Analysis			Multivariable Analysis		
		OR	95% CI	P Value	^a^ OR	95% CI	^a^ *p* Value
Gender							
	Male	—	—	—	—	—	—
	Female	2.042	1.425~2.927	<0.001	3.914	2.309~6.634	<0.001
Age (years)		1.020	1.006~1.034	0.005	1.025	1.002~1.048	0.034
Occupation							
	Teacher/cadre/medical staff	—	—	—	—	—	—
	Farmer/manual worker	3.392	2.207~5.214	<0.001	2.129	1.125~4.031	0.020
	Student/catering & food industry/commercial service/housework & unemployed/self-employed/other	0.660	0.409~1.064	0.088	0.410	0.208~0.808	0.010
Education level							
	College or above	—	—	—	—	—	—
	High school or below	2.591	1.802~3.726	<0.001	1.286	0.715~2.313	0.401
Marital status							
	Married	—	—	—	—	—	—
	Unmarried/divorced	2.528	1.519~4.206	<0.001	9.961	4.478~22.155	<0.001
Household registration							
	Within province	—	—	—	—	—	—
	Outside province	3.363	2.069~5.468	<0.001	2.542	1.333~4.850	0.005
Monthly income (RMB)							
	<5000	—	—	—	—	—	—
	5000–9999	0.532	0.352~0.805	0.003	1.206	0.639~2.276	0.563
	≥10,000	0.460	0.263~0.804	0.006	0.806	0.369~1.763	0.590
Type of medical insurance							
	Public expense/new rural cooperative medical scheme	—	—	—	—	—	—
	Employee/basic medical insurance	0.642	0.435~0.946	0.025	1.961	1.126~3.415	0.017
Chronic diseases							
	No	—	—	—	—	—	—
	Yes	2.092	1.395~3.318	<0.001	2.495	1.451~4.288	0.001
Diabetes duration (years)							
	≤5	—	—	—	—	—	—
	>5	1.363	0.948~1.960	0.094	1.038	0.617~1.746	0.889
Smoking frequency							
	Never	—	—	—	—	—	—
	Occasionally	0.490	0.318~0.754	0.001	0.940	0.540~1.635	0.826
	Frequently	5.098	3.143~8.268	<0.001	11.896	5.555~25.477	<0.001
Alcohol drinking frequency							
	Never	—	—	—	—	—	—
	Occasionally	0.577	0.365~0.912	0.019	0.683	0.368~1.264	0.225
	Frequently	2.330	1.466~3.703	<0.001	1.636	0.791~3.386	0.184

^a^ OR and ^a^ *p* denote the adjusted Odds Ratio and P-value from multivariable logistic regression.

## Data Availability

The data presented in this study are available on request from the corresponding author. The data are not publicly available due to privacy and ethical restrictions.

## Supplementary Materials

The following supporting information can be downloaded at https://www.mdpi.com/article/10.3390/healthcare14152315/s1.
